# Recombinant snakebite antivenoms: A cost-competitive solution to a neglected tropical disease?

**DOI:** 10.1371/journal.pntd.0005361

**Published:** 2017-02-03

**Authors:** Andreas H. Laustsen, Kristoffer H. Johansen, Mikael Engmark, Mikael R. Andersen

**Affiliations:** 1 Department of Biotechnology and Biomedicine, Technical University of Denmark, Kongens Lyngby, Denmark; 2 National Veterinary Institute, Technical University of Denmark, Kongens Lyngby, Denmark; 3 Department of Bio and Health Informatics, Technical University of Denmark, Kongens Lyngby, Denmark; Liverpool School of Tropical Medicine, UNITED KINGDOM

## Abstract

Snakebite envenoming is a major public health burden in tropical parts of the developing world. In sub-Saharan Africa, neglect has led to a scarcity of antivenoms threatening the lives and limbs of snakebite victims. Technological advances within antivenom are warranted, but should be evaluated not only on their possible therapeutic impact, but also on their cost-competitiveness. Recombinant antivenoms based on oligoclonal mixtures of human IgG antibodies produced by CHO cell cultivation may be the key to obtaining better snakebite envenoming therapies. Based on industry data, the cost of treatment for a snakebite envenoming with a recombinant antivenom is estimated to be in the range USD 60–250 for the Final Drug Product. One of the effective antivenoms (SAIMR Snake Polyvalent Antivenom from the South African Vaccine Producers) currently on the market has been reported to have a wholesale price of USD 640 per treatment for an average snakebite. Recombinant antivenoms may therefore in the future be a cost-competitive alternative to existing serum-based antivenoms.

## Author summary

Given the medical importance of snakebite envenoming and the current shortage of antivenoms in sub-Saharan Africa, technological advances in antivenom development and production are needed. One of the avenues that could be taken involves the use of recombinant antivenoms based on oligoclonal mixtures of human IgG antibodies, since these may have the benefits of being compatible with the human immune system and their production is independent on animal immune systems and venom procurement. However, an important aspect of introducing recombinant antivenoms to the clinic is their cost of production given that snakebite victims are often poor rural workers living in remote parts of the tropical parts of the developing world. Here, we aim to provide cost estimates of recombinant antivenom manufacture with special focus on snakebite envenoming in sub-Saharan Africa. Our results indicate that recombinant antivenoms in the future will indeed be cost-competitive compared to existing animal-derived serum-based antivenoms. Furthermore, we outline different manufacturing strategies and suggest the use of caprylic acid precipitation as a low cost purification method following cultivation of CHO cells for antibody expression due to its use in current antivenom manufacture.

## Introduction

The global disease burden from snakebite envenoming is massive, and particularly affecting poor rural tropical areas in Africa, Asia, Oceania, and Latin America [1]. The incidence of envenoming is estimated to be in the order of 2–3 million per year, resulting in more than 100,000 deaths [2,3]. Although animal-derived antisera remain the cornerstone of snakebite therapy [4], biotechnological advances are driving the emergence of different antivenom formats based on human or camelid antibody scaffolds [5,6], which in the future may pave the way for recombinant oligoclonal mixtures of antivenom antibodies [7]. The potential benefits of recombinant antivenoms for treatment of snakebite envenoming include higher potency and fewer side effects (serum sickness and anaphylaxis is not uncommon from animal-derived antisera) due to the possibility of producing fully human antibody formats specifically targeting the medically relevant snake venom toxins [6,8]. In the production of serum-based antivenoms, the therapeutically relevant antibodies targeting snake venom toxins cannot easily be separated from the therapeutically irrelevant antibodies targeting other targets (e.g. bacteria or vira that the immunized animal has encountered during its life. In contrast, recombinant antivenoms may be produced with a significantly higher concentration of therapeutically active antibodies than current serum-based antivenoms, which are known to only contain between 5–36% specific antibodies directed against venom components [9–11]. However, lack of cost-competitive production of antivenom antibody mixtures remains a critical hurdle against making such medicines widely available in poor rural regions of the developing world.

Four families of venomous snakes exist (Elapidae, Viperidae, Atractaspididae, and Colubridae), of which the elapids (such as mambas, cobras, and coral snakes) and viperids (such as rattlesnakes and other vipers) are responsible for the vast majority of envenomings [12]. Generally, viperid venoms are cytotoxic, hemotoxic, and occasionally myotoxic, whereas elapid venoms primarily cause systemic neurotoxicity [12]. The difference in clinical manifestations of viper and elapid venoms stem from the different families of toxins in the snake venom. Further, some of the venom toxins act independently of each other, whereas for others the toxicity is potentiated via toxin synergism [13]. Neurotoxins must first pass the systemic circulation before reaching the relevant targets in the central nervous system and are therefore typically rather small in size. In contrast, toxins which induce tissue damage, including proteases, cytotoxins, and myotoxins, are larger proteins which primarily exert their destructive effects at the site of the bite. This difference in site of action for different toxins means that antivenoms against locally-acting toxins need to be able to reach distal sites and deep tissue [14], whereas rapid distribution in the circulatory system may be sufficient for effective delivery of antivenoms against systemic toxicity.

**Fig 1:**
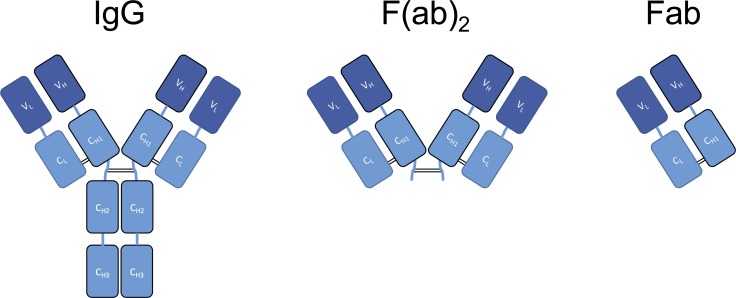
Overview of the three antibody formats commonly used in serum-based antivenoms. IgG is the entire native immunoglobulin G, whereas the formats F(ab)_2_ and Fab are obtained by enzymatic cleavage in the hinge region with pepsin or papain, respectively.

Currently, animal-derived snakebite antivenoms are manufactured in three different structural formats: IgG-based, F(ab)_2_-based, and Fab-based [6,14] (see Fig 1). F(ab)_2_ and Fab-based are generated with the intention of creating improved safety profiles through the removal of the Fc region of the animal-derived IgG antibodies by treatment with pepsin or papain during the manufacturing process [7]. Fab-based antivenoms have been shown to better reach and neutralize toxins in deep tissue than IgG-based antivenoms, but it comes at a cost of reduced serum half-life [14]. In comparison, F(ab)_2_-based antivenoms have pharmacokinetic properties somewhat in between IgG-based antivenoms and Fab-based antivenoms [14]. Therefore, different antivenom formats have different benefits and drawbacks. The IgG format may represent a more optimal solution for targeting systemically acting toxins, whereas the Fab format may potentially be more ideal against locally acting toxins. Other formats have been investigated, including single-chain variable fragments (scFv) and camelid single-domain antibodies (V_H_H fragments) [5–7], but none of these are have yet been tested in the clinic.

The standard method for purification of IgGs from the plasma of immunized animals is based on caprylic (octanoic) acid precipitation [15]. This method is inexpensive and robust, but also leaves unwanted traces of caprylic acid in the final product. In order to increase the purity of antivenoms, additional chromatographic steps can be applied, although this leads to lower production yield and significantly higher production cost [16]. Caprylic acid precipitation of IgGs from plasma should be compared to recombinant mAb production, where the standard methods of purification are based on affinity chromatography using protein A capture. The advantage of protein A chromatography is that it leads to a purer product. However, the method is also significantly more expensive, and the resins used for affinity binding of antibodies have a limited number of uses [17,18], typically 150–200 (personal communication with Anne Tolstrup, Biogen (Denmark) A/S).

**Fig 2:**
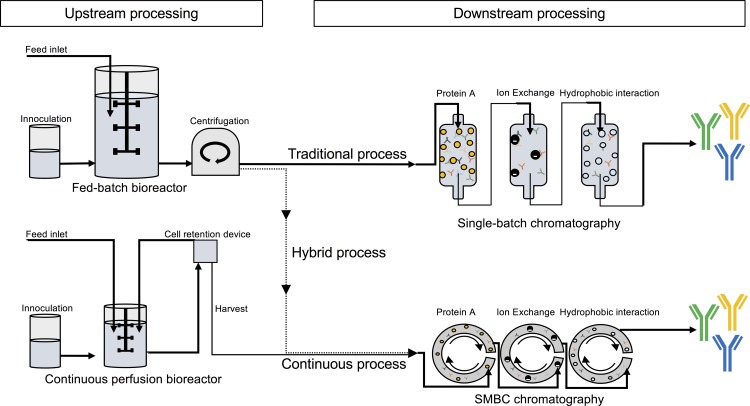
Overview of the three different antibody manufacturing process strategies: In the fed-batch process, nutrients for the CHO cells are supplied for a complete cultivation process followed by harvest and purification of the entire batch by single-batch chromatography. In the continuous perfusion process, cells are retained while the growth medium containing the antibodies is continuously substituted with fresh medium in a perfusion reactor. The used media undergoes simulated moving bed chromatography (SMBC), where the chromatographic processes are conducted in a continuous process as described in [22]. In the hybrid process, cultivation is performed in a fed-batch reactor followed by SMBC instead of single-batch chromatography.

For large-scale production of monoclonal recombinant antibodies, Chinese Hamster Ovary (CHO) cell-based expression systems are most common [19], partially due to their ability to produce glycosylation patterns similar to human patterns. CHO cell expression is traditionally performed in a fed-batch process (see Fig 2), where nutrients for the CHO cells are supplied for a complete manufacturing process followed by harvest of the entire batch. The antibodies from this batch are then purified by one or more chromatographic purification steps. Other cost-competitive production processes, such as hybrid and continuous perfusion processes (see Fig 2) are emerging [20]. In the hybrid process, cultivation is performed in a fed-batch reactor followed by continuous or semi-continuous purification of the produced antibodies. In the continuous perfusion process, the cultivated cells are retained while the growth medium containing the antibodies is continuously substituted with fresh medium in a perfusion reactor. The used medium undergoes a continuous or semi-continuous purification process in order to isolate the antibodies [20,21] (see Fig 2). In the continuous perfusion process, several companies in the industry are employing continuous chromatographic procedures, such as Simulated Moving Bed Chromatography (SMBC) (personal communication with Mads Laustsen, Symphogen A/S). This procedure allows for efficient use of chromatographic media as well as it yields higher output of purified product [22]. In the work presented here, we base our calculations on hybrid and continuous perfusion processes using SMBC as downstream process [21]. In addition, we compare the results of these calculations with a manufacturing setup, where the hybrid and continuous processes employ caprylic acid precipitation as purification method [20], since this downstream process poses as a cost-competitive alternative that is already employed in existing antivenom manufacture.

**Fig 3:**
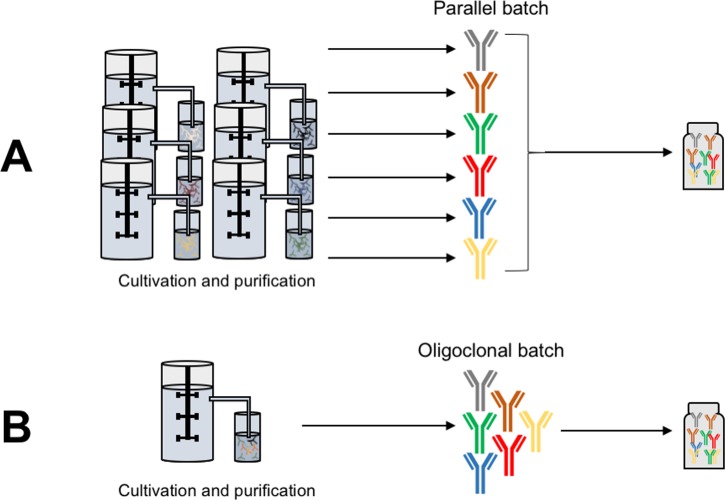
Schematic representation of two different strategies for recombinant expression of antibody mixtures. A) Mixing of batches: Antibodies are produced by monoclonal expression in different bioreactors, followed by individual purification, after which they are mixed to yield the final oligoclonal antivenom product. B) Oligoclonal expression: Antibodies are produced by oligoclonal expression in one bioreactor containing different cell lines. Purification can then be performed on the mix of antibodies, providing the final oligoclonal antivenom.

In later years, a growing interest in producing mixtures of monoclonal antibodies has emerged based on an expectation that improved antibody-based therapies can be obtained by targeting more than one target in multiple different indications. Two different strategies can be employed to produce such antibody mixtures: 1) Mixing of individually produced parallel batches of each monoclonal antibody (Fig 3A) [23–25] and 2) oligoclonal expression of antibodies in a single batch (Fig 3B), exemplified by Merus’ Oligoclonics technology [26] and Symphogen’s Sympress technology [23,25]. In the Oligoclonics technology, a single cell line is transfected with a shared light chain and 2–3 different heavy chains giving rise to several different specificities. In the Sympress technology, a range of clonal cell lines each expressing different antibodies are mixed together in a single batch in order to produce a mix of a desired number of specific antibodies. The Oligoclonics technology limits the number of different specificities as the single cell line expression system gives rise to stability challenges with higher numbers of different heavy chains [27]. The Sympress technology on the other hand allows for any number of different specificities as each antibody specificity is produced by a separate cell line [23,25].

A single monoclonal antibody is unlikely to be able to effectively neutralize a snake venom, given the complexity of snake venoms, which each contain numerous different toxins from different protein families [8]. Instead, mixtures of antibodies will be needed in order to neutralize most of the medically relevant snake venom toxins [6–8,28]. Moreover, it is desirable to have polyvalent antivenoms that target the venom toxins from more than just a single snake species, since it is not always possible for clinicians to identify the perpetrating snake species with certainty. Therefore, a clear need exists for technologies enabling cost-competitive production of oligoclonal antibody mixtures, if recombinant antivenoms are to be introduced. Actual proof of concept studies on the production of recombinant antivenoms have never been reported. However, sufficient knowledge from related areas within antibody expression and CHO cell cultivation exists to assess the utility and cost-competitiveness of future recombinant antivenoms. Here, we provide an estimate of the production costs of oligoclonal recombinant antivenoms based on industry data and discuss the implications that novel antibody expression technologies may have on future snakebite envenoming therapy. Antivenoms are likely to be produced with the aim of being able to neutralize most of the medically relevant snakebites in a particular region of the world, but not the entire world. Therefore, we base our present estimates on a hypothetical polyvalent antivenom that could be used to treat the approximately one million snakebite envenomings occurring each year in sub-Saharan Africa, as such pan-African antivenoms derived from animal serum already exist for comparison.

## Methods

### Estimation of the minimum need for therapeutically active antibodies in antivenom in sub-Saharan Africa

The estimate of the minimum need for active antibodies (active pharmaceutical ingredient, API) to neutralize snakebite envenoming is critical for establishing the maximum cost of production for a recombinant antivenom, as this cost is highly dependent on the scale of production. Here, the minimum need for active antibodies is based on the number of snakebite envenomings in sub-Saharan Africa (approximately one million victims) [2], the average number of vials needed to treat an envenoming (for the representative SAIMR Snake Polyvalent Antivenom from the South African Vaccine Producers), which is estimated to be between 6 and 10 vials per treatment [29], the volume and protein content of this antivenom (10 mL and 172 mg/mL) (as measured by their absorbance at 280 nm on a NanoDrop 2000c instrument, Thermo Scientific) [30], and the lowest estimate (5%) of the percentage of venom-recognizing antibodies in an antivenom (ranging between 5% and 36%) [9–11]. In fact, the amount of therapeutically active antibodies is likely to be even lower, since not all venom components are medically relevant, yet still induce an immune response during the animal immunization process. Also, we assume that antibodies produced recombinantly are equipotent (mol-to-mol) to the therapeutically active antibodies found in current antivenoms, and we take the difference in molecular weight between IgG molecules and F(ab)_2_ fragments into account in the calculation of the minimum need of antibodies. Finally, we do not take into account any amount of antivenom that may be discarded due to expiration.

### Cost of Goods Manufactured (COGM) per gram for oligoclonal antibody production

The cost of recombinant monoclonal antibodies is highly dependent on the manufacturing approach and the scale of production. The Cost of Goods Manufactured of Active Pharmaceutical Ingredient (COGM_API_) has been analyzed extensively on several occasions [20,31,32]. Production costs of monoclonal antibodies range from 36 USD/g to 1500 USD/g depending on the manufacturing facilities used [20,33] and when the process was developed (personal communication with Anne Tolstrup, Biogen (Denmark) A/S). We base our calculations on the Sympress technology, as this has been successfully employed in the development and production of several oligoclonal antibody-based therapies that have entered clinical trials (http://www.symphogen.com/pipeline). The COGM_API_ of recombinant oligoclonal antibodies is estimated to be 0–10% higher than the production cost of conventional mAbs [25]. In our analysis, four manufacturing situations (annual production of 100 kg_API_, 200 kg_API_, 500 kg_API_, and 1,000 kg_API_), based on reported cost estimates for fed-batch, hybrid, and continuous perfusion processes [31] were employed to estimate COGM_API_ for oligoclonal antibody production. The reported cost estimates in [31] are calculated based on SMBC as the purification process, using the most realistic assumptions for fed-batch, hybrid, and continuous perfusion, where the fed-batch reactors are used multiple times to obtain titers of 3 g/L, and where continuous perfusion is performed with a cell specific perfusion rate (CSPR) of 0.05 nL per cell^-1^ d^-1^ with a titer of 0.4 g/L [21].

### Cost of Goods Manufactured (COGM) per treatment for oligoclonal recombinant antivenoms

**Fig 4:**
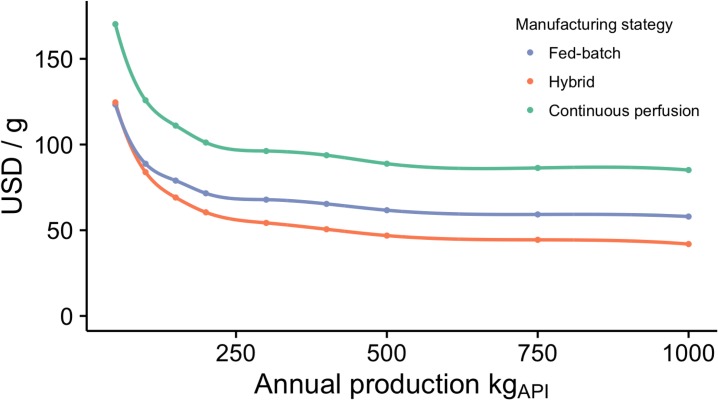
Estimation of Cost of Goods Manufactured (COGM) per gram for oligoclonal antibody production at different scales of production for three different manufacturing strategies, Fed-batch, hybrid, and continuous perfusion (all using chromatography as purification method, see Fig 2), based on cost estimates from [25,31].

To estimate the COGM_API_ for a snakebite treatment using oligoclonal recombinant antivenoms at a scale of 500 kg_API_, direct costs of 62 USD/g (fed-batch), 47 USD/g (hybrid), and 89 USD/g (continuous perfusion) (determined in the previous section) were employed (also see Fig 4). Estimates were based on two different relative amounts (5% and 20%) of therapeutically active antibodies present in current antivenoms. The number of vials per average treatment for the SAIMR Snake Polyvalent Antivenom from the South African Vaccine Producers was based on data published in [29]. Vial volume and protein concentration are equal to those given above. Finally, to estimate the Cost of Goods Manufactured for the final drug product (COGM_FDP_) the cost of formulation and packaging was (in discussion with Mads Laustsen, Symphogen A/S) estimated to be 5 USD/vial, which is five times the cost reported for current antivenoms in [34], and the upper limit for the concentration of active antibodies in 10 mL vials with recombinant oligoclonal antivenom was set to 100 mg/mL. For comparison, we performed cost estimates based on an alternative set of numbers for production of oligoclonal recombinant antivenom using caprylic acid precipitation as the purification method for hybrid and continuous perfusion processes at a scale of 500 kg_API_. Here, we applied COGM_API_ from [20] for commercial production and added 10% for oligoclonal production [25]: 46 USD/g for fed-batch cultivation followed by single-batch chromatography, 33 USD/g for hybrid (fed-batch cultivation followed by continuous precipitation-based purification), and 42 USD/g for continuous perfusion followed by continuous precipitation-based purification.

## Results and discussion

### Minimum antivenom need in sub-Saharan Africa

The unit cost of recombinant antibody production is highly dependent on the scale of antibodies produced [31,35]. To estimate the order of magnitude of the amount of antibodies needed to treat all snakebites in sub-Saharan Africa, we disregarded the fact that many antivenoms may not be used for effective therapeutic treatment of victims due to expiration or loss during the supply chain. Thus, we made the assumption that manufacturing volume equals need, which may not be the case in reality. Also, we only considered the therapeutically active antibodies in current antivenoms, which are likely to be no more than the amount of antibodies in antivenoms that are able to recognize snake toxins: 5% to 36% [9–11], since there is no reason to include ineffective antibodies, when producing antibodies using a recombinant approach. In our assessment of the sub-Saharan African need, we employed the lowest average number of vials (6 vials, 10 mL/vial) reported for the SAIMR Snake Polyvalent Antivenom from the South African Vaccine Producers [29] and the lowest estimate of the content of therapeutically active antibodies in this antivenom, being 5%. Based on these assumptions, we provide the conservative estimate that the sub-Saharan African need for therapeutically active antivenom antibodies to be at least 500 kg_API_ per year. This number does not represent a precise estimation of the actual need, however, it provides a lower estimate of the magnitude required for production of antibodies for recombinant antivenoms, which may further be used to estimate the COGM_API_ (USD/gram) for the manufacture of recombinant antibodies. In reality, the real need may be in the order of 1 to 2 ton_API_ per year (if e.g. the assumed content of therapeutically active antibodies is assumed to be 10–20% instead of only 5% for the SAIMR Snake Polyvalent Antivenom from the South African Vaccine Producers). However, increasing the production volumes above 250 kg_API_ per year has limited influence on COGM_API_ [31] (disregarding any possible capital investment needed in reality to modify production facilities), see Fig 4.

### Cost of Goods Manufactured (COGM) for oligoclonal recombinant antivenoms

In our assessment of the COGM_API_ for oligoclonal recombinant antivenoms produced via a fed-batch, hybrid, and continuous perfusion process, we employed cost estimates provided by industry [31] according to a scale of production in the range of 500 kg_API_ per year is estimated in the section above (see Fig 4 for cost as a function of scale of production). At this scale, the COGM_API_ for production of oligoclonal antibody mixtures is estimated to 62 USD/g for a fed-batch process, 47 USD/g for a hybrid process, and 89 USD/g for a continuous perfusion process in agreement with similar cost estimates reported in literature [20,25,31,36,37].

**Fig 5:**
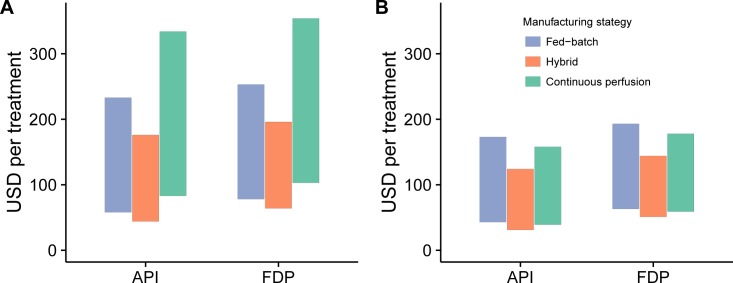
Estimation of Cost of Goods Manufactured (COGM) per snakebite treatment for oligoclonal antibody production at a production scale of 500 kg_API_ for three different manufacturing strategies: Fed-batch, hybrid, and continuous perfusion. A) Cost estimated based numbers from [31], employing SMBC as purification method for the hybrid and continuous perfusion processes. B) Cost estimated based numbers from [20], employing continuous caprylic acid precipitation as purification method for the hybrid and continuous perfusion processes instead of continuous SMBC. API: Active Pharmaceutical Ingredient. FDP: Final Drug Product.

To compare the COGM_API_ per treatment, we here employed both a low estimate (5% of the antivenom antibodies are active) and an estimate that we consider more realistic (20% of the antivenom antibodies are active) based on numbers from [9–11]. In these calculations, we assumed that the average snakebite envenoming requires 8 vials (10 mL/vial) of SAIMR Snake Polyvalent Antivenom [29]. As it can be seen in Fig 5A, the COGM_API_ per treatment for oligoclonal recombinant antivenoms at a scale of 500 kg_API_ estimated to be between USD 58–233 in fed-batch mode, USD 44–176 in hybrid mode, and USD 83–334 in continuous perfusion mode based on the low and high estimates of the content of therapeutically active antibodies in an antivenom.

In order to estimate the Cost of Goods Manufactured for the Final Drug Product (COGM_FDP_), which includes formulation and packaging, we used a cost estimate of 5 USD/vial. We further assumed that each 10 mL vial could be formulated with an antibody concentration of up to 100 mg/mL, but that for administration purposes an average envenoming should be treated with at least 4 vials. This would allow a physician to easily administer less antivenom for milder envenomings requiring a dose lower than average. Since much fewer antibodies are needed in a recombinant antivenom compared to traditional antisera, as only the therapeutically active antibodies are manufactured, these criteria had the effect that treatments with all antibody formats would meet the minimum requirement of 4 vials for formulation, adding 20 USD to the COGM of treatment. The COGM_FDP_ per treatment is therefore estimated to USD 78–253 in fed-batch mode, USD 64–196 in hybrid mode, and USD 103–354 in continuous perfusion mode (see Fig 5A). The fairly broad estimates for each individual situation derives from the uncertainty of how many therapeutically active antibodies actually exist in the existing SAIMR Snake Polyvalent Antivenom. In these estimates, we employed a percentage between 5% and 20%, which corresponds to a 4-fold increase from the lowest estimate to the highest. More knowledge and better understanding of the therapeutic content of existing antivenoms would be useful to narrow down the cost estimates presented here. Even more desirable, however, would be to have proof of concept recombinant antivenoms with a defined set of discovered and tested human monoclonal antibodies upon which better cost estimates could be performed. As effective doses of individual antibodies in real case scenarios may span 1–2 log units, having such a defined set of antibodies with specific ED_50_s would allow for bottom-up calculations based on optimal molecular ratios between antibodies (removing the need for assuming equipotency (mol-to-mol) of antiserum-based antibodies and recombinantly produced antibodies, see Methods). In real case scenarios, minimum requirements for antibody efficacy and ease of expression should be imposed as selection criteria to ensure that the antibodies selected for a recombinant antivenom are compatible with oligoclonal expression methods (e.g. Sympress or Oligoclonics).

Since caprylic acid precipitation is routinely employed in the manufacture of current antivenoms [15], a comparison of the COGM_FDP_ per treatment for oligoclonal recombinant antivenoms produced using continuous caprylic acid precipitation for the hybrid and continuous perfusion processes was performed. Based on numbers from [20,25], the COGM_API_ was in this case estimated to 46 USD/g for fed-batch cultivation followed by single-batch chromatography, 33 USD/g for hybrid (fed-batch cultivation followed by continuous precipitation-based purification), and 42 USD/g for continuous perfusion followed by continuous precipitation-based purification. These cost estimates based on the application of caprylic acid precipitation for the hybrid and continuous process are significantly lower than the estimates based on processes employing chromatography. Using these lower cost estimates therefore leads to a significant lowering of the COGM_API_, estimated to USD 43–173 for fed-batch mode, USD 31–124 for hybrid mode, and USD 39–158 for continuous perfusion mode. When including 4 vials for formulation, the COGM_FDP_ is estimated to USD 63–193 for fed-batch mode, USD 51–144 for hybrid mode, and USD 59–178 for continuous perfusion mode (see Fig 5B). Our results may therefore point towards the applicability of caprylic acid precipitation for manufacture of recombinant antivenoms due to its lower cost and compliance with current antivenom manufacture. In general, since we estimate the cost estimates on oligoclonal expression by adding 10% to the COGM_API_ for oligoclonal antibodies compared to monoclonal antibodies, the cost estimates presented here subtracted these 10% may also be valid for the production of recombinant antivenoms based on monoclonal antibodies against animal venoms, where venom toxicity can be abrogated by targeting a single toxin (or more toxins with a cross-reactive antibody).

The COGM estimates (approx. USD 60–350 per treatment for processes using chromatography as purification method and approx. USD 50–190 per treatment for processes using caprylic acid precipitation as purification method) presented here compare favorably with the wholesale cost of SAIMR Snake Polyvalent Antivenom (USD 640 per treatment) [29]. Although the wholesale cost may not adequately reflect the COGM_FDP_ for SAIMR Snake Polyvalent Antivenom, our COGM_FDP_ estimates for recombinant production indicate that even with a fairly high margin for distribution, sales, and profit (45–92% depending on manufacturing strategy and purification method), that snakebite envenoming treatments based on recombinant antivenoms could be cost-competitive with current serum-based antivenoms.

In this feasibility study, we focus on the production of IgG-based antibodies, since other antibody formats, such as Fab, scFv, or V_H_H would not be produced in CHO cells, but rather in prokaryotic systems (personal communication with Mads Laustsen, Symphogen A/S). We therefore do not intend to provide an in-depth discussion on any therapeutic or pharmacokinetic benefits that some antibody formats may have above others. Some of these differences include the bivalency of IgG and F(ab)_2_ and the prolonged half-life of IgG compared to both F(ab)_2_, Fab, scFv, and V_H_H fragments [6,14,38,39]. The shorter half-life of F(ab)_2_s, Fabs, scFvs, and V_H_Hs makes it likely that larger doses of these more rapidly degraded formats are needed to effectively neutralize all venom toxins in a snakebite envenoming. Also, even for systemically acting neurotoxins that are able to reach their targets within minutes, a delayed release of toxins from the bite site may occur over a period of days, demanding either repeated doses or longer serum half-life of the antivenom [40]. On the other hand, smaller fragments may possibly penetrate faster into distal tissue, where venom toxins are initially located following a snakebite, possibly providing therapeutic benefits to the use of smaller fragments. It is likely that Fabs, scFv, or V_H_H-based recombinant antivenoms could be produced in large scale at an even lower cost using prokaryotic systems that do not require expensive media, and which have a high growth rate and productivity. A COGM analysis comparing the different formats and taking the molecular and pharmacokinetic differences into account is, however, beyond the scope of this study as we deem it to be too speculative, given the large amount of assumptions that would need to be made. Since the majority of current antivenoms (including the SAIMR Snake Polyvalent Antivenom) are based on the F(ab)_2_ format [6], which has a comparable molecular size and bivalence, the COGM estimates presented here may indeed provide a rough assessment of the relative cost-competitiveness of IgG-based antivenoms. Moreover, the human IgG format with its long half-life has never been thoroughly explored in the field of antivenom, and it may indeed provide additional benefits such as lower administration doses needed for the treatment of a snakebite envenoming. The data presented here therefore warrant further research into the development of recombinant antivenoms. Additionally, a need presents itself for discussing how to handle regulatory affairs, when a switch from conventional antisera (which are defined as “blood products”) to recombinant antivenoms (probably to be defined as “biopharmaceuticals/biologics”) occurs.

As a final remark, the cost of recombinant antibody production has been hypothesized to possibly reach as low levels as 10–20 USD/g in the longer term [33,36,41]. Although reaching such a low cost of production may be years into the future and require a larger scale than what is relevant for antivenoms, it may indicate that the cost of recombinant antivenoms in the future may be even lower than what is estimated here.

## Conclusions

Based on industrial cost estimates of oligoclonal expression of antibodies at large scale and the estimated need for therapeutically active antibodies to treat the one million snakebite envenomings occurring each year in sub-Saharan Africa, we estimate that recombinant pan-African antivenoms could be produced with a COGM_API_ of 62 USD/g (fed-batch), 47 USD/g (hybrid process), and 89 USD/g (continuous perfusion), when chromatography is used as purification method, and 46 USD/g (fed-batch), 33 USD/g (hybrid process), and 42 USD/g (continuous perfusion), when caprylic acid precipitation is used as purification method for the hybrid and continuous perfusion process. This translates into an approximate Cost of Goods Manufactured for the IgG-based final drug product including vials and formulation (COGM_FDP_) between approx. USD 60–350 per treatment (for processes using chromatography as purification method) and approx. USD 50–190 per treatment (for processes using caprylic acid precipitation as purification method) for treating an average snakebite envenoming that would normally require 8 vials of SAIMR Snake Polyvalent Antivenom from the South African Vaccine Producers [29]. These estimates compare favorably with previously reported costs of treatment ranging from USD 56 to 640 for current serum-based antivenoms [29], leaving a large margin (45–92%) for formulation, distribution, sales, and profit when comparing with the golden standard antivenom, SAIMR Snake Polyvalent Antivenom.

The data-supported cost estimates presented here may provide better insight into the economics of recombinant antivenom production and help guide decision processes on what technological platforms future antivenoms should be built on. Our estimates demonstrate the cost-competitiveness of the production of recombinant antivenoms, which may provide incentive to more researchers to engage in the development and testing of such novel therapies against snakebite envenoming.
